# Combination of ultrasound guided superficial cervical plexus block and local infiltration for oromaxillofacial surgeries: a case series

**DOI:** 10.3389/fonc.2024.1412062

**Published:** 2024-11-07

**Authors:** Hao-ran Zhao, Jian-shuai Hao, Ling-fa Xue, Jin-ze Zhao, Yi-chen Wang, Wen-lin Xiao

**Affiliations:** ^1^ Department of Oral and Maxillofacial Surgery, Affiliated Hospital of Qingdao University, Qingdao, Shandong, China; ^2^ School of Stomatology, Qingdao University, Qingdao, Shandong, China; ^3^ Department of Anesthesiology, Affiliated Hospital of Qingdao University, Qingdao, Shandong, China; ^4^ School of Stomatology, Nanjing Medical University, Nanjing, Jiangsu, China

**Keywords:** elderly patients, interventional ultrasonography, superficial cervical plexus block, local infiltration anesthesia, oral and maxillofacial surgery

## Abstract

**Introduction:**

When elderly patients have underlying diseases combined with oromaxillofacial diseases requiring surgical treatment, the application of conventional general anesthesia (GA) for oromaxillofacial surgical diseases has become a risk due to underlying disease reasons. The objective of this study was to evaluate the efficacy and safety of ultrasound-guided superficial cervical plexus block (SCPB) anesthesia combined with local infiltration anesthesia (LIA) for partial oral and maxillofacial surgery (OMFS) in patients who with risk for GA due to underlying disease.

**Methods:**

The clinical data of 7 high risk patients with OMFS treated with SCPB anesthesia combined with LIA were retrospectively analyzed. All seven surgeries were performed on one side of the neck. All patients were given ultrasound-guided SCPB anesthesia by the same anesthesiologist, LIA by the same surgeon, and surgery was performed under continuous Electrocardiogram (ECG) monitoring.

**Results:**

Seven patients had stable vital signs and no significant postoperative complications. The results of this study indicated that SCPB anesthesia combined with LIA is a safe and effective anesthesia technique with a high success rate and patient tolerance.

**Discussion:**

For patients with OMFS who have a risk for GA due to underlying diseases, ultrasound-guided cervical superficial plexus block anesthesia combined with LIA is a safe and effective alternative to conventional GA.

## Introduction

As the global population continues to age and the associated social and economic costs rise, the issue of ageing has become a significant concern on a global scale (1). China’s aging population is a significant trend in social development. By 2035, over 400 million people in China will be aged 60 and above, making up more than 30% of the total population. The elderly population in China is expected to peak around 2050 (2). Modern medicine is facing challenges due to the increasing elderly population and high rates of age-related diseases (3).

Given the diminished function of tissues and organs in the elderly population, their immune function and tolerance are compromised, underscoring the necessity of selecting appropriate and effective anesthesia methods for elderly surgical patients (4). Anesthesia is not only an important basis to ensure the orderly implementation of surgical programs, but also different anesthesia methods have different effects on postoperative recovery of elderly patients (4).

General anesthesia (GA) is a simple and relatively safe method used in modern medicine to achieve surgical anesthesia, and oral and maxillofacial surgery (OMFS) is no exception. However, GA also has many disadvantages: high economic costs, the need for a large number of trained personnel, high morbidity, mortality and equipment requirements. GA can reduce intraoperative pain and improve intraoperative comfort, but its safety remains to be explored due to the large dose of drugs used and the many side effects of anesthetic drugs acting on organs throughout the body. Especially when some elderly patients have poor systemic condition and more underlying diseases, the risk of surgery under GA is greatly increased. In order to help these elderly patients recover their health and give these elderly people a chance for surgical treatment, regional anesthesia may be a feasible method.

Superficial cervical plexus block (SCPB), a type of cervical plexus block anesthesia, is a simple technique that is easy to implement and, in some cases, takes precedence over GA (5). Especially in elderly patients with medical conditions that do not permit GA surgery, the use of regional anesthesia (RA) provides a stress-free anesthesia environment because of its simple technique, low catecholamine release, reduced blood loss due to local vasoconstrictors and sympathetic block, and negligible morbidity with appropriate doses of local anesthetics (6). Halstead first proposed and performed cervical plexus block anesthesia (CPBA) in 1884, after which Kappis described the approach to CPBA (7). Heidenhein introduced the lateral approach technique for CPBA, which Labat popularized (8). SCPB technique is suitable for RA in the area around the jaw, neck and ear. Studies have reported that this is a potentially valuable anesthesia technique for patients with conditions such as ear lobe laceration, submandibular abscesses, and injuries to the mandibular region or neck (9). SCPB has been used in various disciplines, such as thyroidectomy, carotid endarterectomy, vocal cord surgery, and cervical pain syndrome (7–9). To date, its applications in oral and maxillofacial surgery (OMFS) have included surgical drainage of abscesses in the peri mandibular area, removal of superficial lesions in the peri ear or neck area, and skin sutures in the corresponding area (10).

Perisanidis et al. first applied ultrasound-guided middle and deep CPBA in OMFS patients (11). Later, new ultrasound-guided SCPB was used in emergency anesthesia and analgesia (12). Inspired by previous studies, for elderly patients with underlying diseases requiring OMFS, GA carries a risk for these patients. We successfully performed surgery on 7 patients with ultrasound-guided SCPB anesthesia combined with local infiltration anesthesia (LIA). The aim of this study was to evaluate the feasibility, safety, and efficacy of SCPB as an alternative to GA in elective cases in the practice of OMFS and to analyze complications related to the procedure.

## Patients and methods

The study was approved by the Ethics Committee of the Affiliated Hospital of Qingdao University (Ethics number, QYFY WZLL 28599). We reviewed the OMFS performed on 7 patients in our department from July 2019 to December 2022 using ultrasound-guided SCPB anesthesia combined with LIA. Informed consent of patients was obtained after correct explanation of surgical procedures.

**Figure 1:**
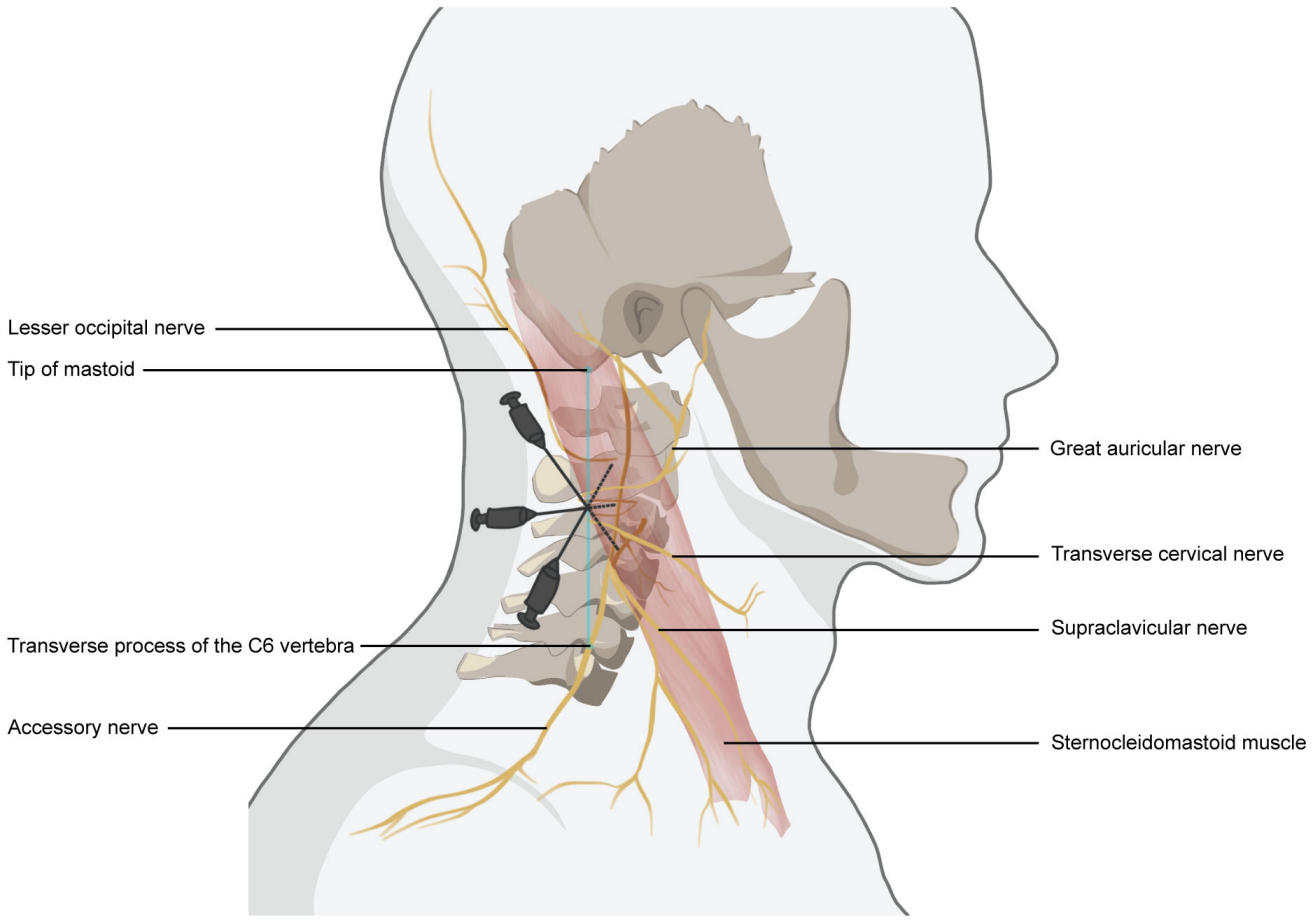
Schematic drawing of superficial cervical nerve block anesthesia. The needle entry point is located at the posterior border of the sternocleidomastoid muscle, at the midpoint of the line connecting the tip of the mastoid process to the transverse process of the C6 vertebra. The depth of needle insertion was 1.0–1.5 cm. Using the “fan” -shaped manner, local anesthetics were injected 2-3 cm above and below the needle insertion site.

**Figure 2:**
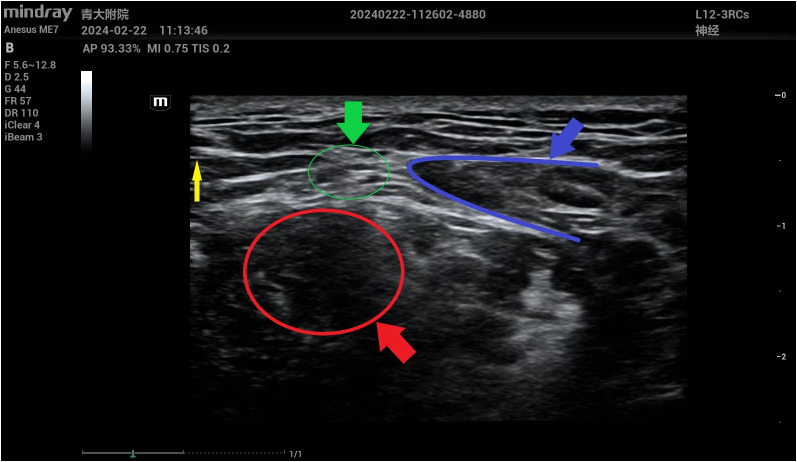
Anatomic marker in ultrasound images of superficial cervical nerve anesthesia under ultrasound guidance. The yellow arrow indicates the puncture needle; The green arrow indicates the superficial cervical plexus; The selected area in the blue frame represents the lateral margin of the sternocleidomastoid muscle; The area selected in the red wire frame indicates the middle scalene muscle.

SCPB is performed by the same anesthesiologist after a thorough evaluation of the regional anatomy with continuous monitoring of all vital signs. All patients underwent SCPB under strict aseptic conditions. The patient was placed in a supine position with the patient’s head slightly turned to the non-anesthetic side. The anesthesiologist’s hand assists the patient to raise his head, outline the sternocleidomastoid muscle and locate the external jugular vein; Locate and mark the posterior border of the sternocleidomastoid muscle in the midpoint between the line joining the mastoid tip with the transverse process of the C6 vertebra; Anesus ME7 (Mindray Ultrasound) was placed on the sternocleidomastoid muscle at the same level as the cricoid cartilage. The lateral margin of sternocleidomastoid muscle, middle scalene muscle, superficial cervical nerve plexus, external jugular vein, internal jugular vein, carotid artery and so on can be seen in ultrasound images. A 5ml injection needle is pushed 2-3 cm up and down the boundary of the sternocleidomastoid muscle, then 0.4% ropivacaine 5ML is injected (**Figures 1**, **2**). Adequacy of the block is determined 10-15 minutes after injection of anesthetic; Objective symptoms are examined after blocking along the SCP distribution and follow-up surgery is performed. Intraoperatively, the surgeon administered an incremental local anesthetic supplement of 1ml of 1% lidocaine if needed.

## Results

Our study included seven patients, three men and four women, aged 69 to 92 years (mean 79.6 years). There were 2 cases of cervical malignant tumor lymph node metastasis, 1 case of branchial cleft cyst, 1 case of submandibular gland malignant tumor, 1 case of submandibular gland benign tumor, and 2 cases of sublingual schwannoma. The operative time ranged from 40 to 120 minutes, with a mean of approximately 89 minutes. Patients were able to lie flat during the whole procedure and were well tolerated. No patients were transferred to GA. No sedative drugs were used in any of the patients (**Table 1**).

**Table 1:** Patient data.

Serial number	age (year)	Gender	Diagnosis	General condition	Type of operation	Mode of anesthesia	complication
1	92	F	Secondary malignant tumor of the neck lymph node metastasis	Bronchitis more than 30 years	SND	SCPB+LIA	None
2	69	M	Secondary malignant tumor of the neck lymph node metastasis	Heart stent implantation more than 10 years	SND	SCPB+LIA	None
3	78	F	Submaxillary gland malignancy	Bronchitis and asthma more than 40 years	Expanded resection of submaxillary gland malignant tumor +SND	SCPB+LIA	None
4	81	M	Hypoglossal schwannoma	Hypertension for more than 30 years	Resection of hypoglossal schwannoma	SCPB+LIA	None
5	79	M	Second branchial cleft cyst	Cerebral thrombosis more than 30 years	Resection of the second branchial cleft cyst	SCPB+LIA	None
6	75	F	Hypoglossal schwannoma	Asthma for more than 5 years	Resection of hypoglossal schwannoma	SCPB+LIA	None
7	83	F	Submaxillary gland benign tumor	Coronary heart disease more than 10 years	Submandibular gland mixed tumor excision	SCPB+LIA	None

Local Infiltration Anesthesia, LIA; Selective neck dissection SND.

Follow-up: The postoperative analgesic effect of SCPB lasted for about 6 hours, and the vital signs remained stable during and after operation, and the postoperative recovery was satisfactory. Satisfaction levels were high, and all seven patients were discharged about one week after surgery. All patients were re-examined every 3 months for 12 months, and there were no significant post-anesthesia complications.

### Case presentation

**Figure 3:**
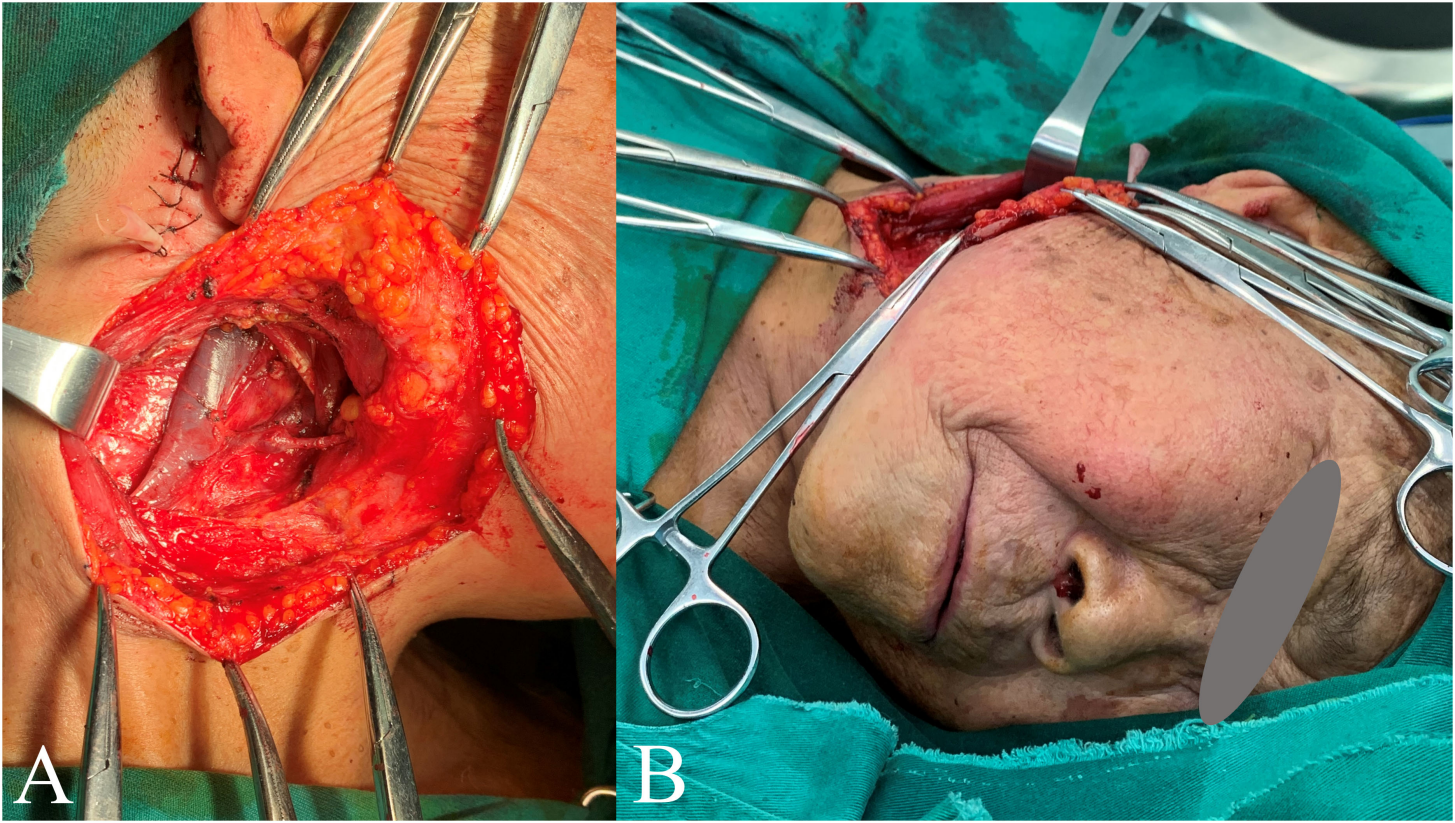
Report case. Panel **(A)** shows the surgical area; Panel **(B)** shows the patient undergoing surgery without intubation, with regional anesthesia of the superficial cervical plexus block combined with local infiltration anesthesia.

A 92-year-old female patient came to our hospital for “right submaxillary painless mass for 2 years” and reported that the right submaxillary mass had grown rapidly in the past six months. The patient underwent surgery 5 years ago for squamous cell carcinoma of the right temporal skin. Physical examination: Patient in autonomous position, conscious. The local tumor was about 3×2×2cm^3^ in size, hard in quality, poor in motion, no adhesion to the skin, and no abnormality in facial nerve function. There was no abnormality in the right temporal cutaneous area. Local biopsy pathology revealed “highly differentiated squamous cell carcinoma (right submaxillary mass)”. Cervical CT showed no lymph node metastasis. The patient suffered from “bronchitis” for more than 30 years and reported chest tightness. Budesonide suspension and terbutaline sulfate were used in daily atomization treatment (once in the morning/day). Preoperative examination was performed after admission, and pulmonary function examination revealed severe diffuse ventilation dysfunction. According to the results of relevant examinations, the anesthesiologist believes that the risk of GA is greater and recommends the patient to undergo local anesthesia. Considering that the effect of local anesthesia alone might not be ideal, the surgeon discussed the case with the anesthesiologist and decided to perform ultrasound-guided SCPB anesthesia combined with LIA. The anesthesiology consultation opinions, surgical plan and risks were informed to the patient and his family members, and the patient and his family members gave informed consent. Then the anesthesiologist performed SCPB anesthesia under ultrasound guidance, and the surgeon performed “expanded resection of right neck malignant tumor + selective neck lymph node dissection” with local anesthesia after fixed point drawing (**Figure 3**). During the operation, the patient was conscious and able to communicate with the surgeon without obstacles. The patient recovered well and was discharged from hospital one week after surgery. Postoperative pathological diagnosis: (right submaxillary mass) highly differentiated squamous cell carcinoma. There was no cancer involvement at each incisal margin. Lymph nodes in area II (0/5) and area III (0/8) were examined.

After discharge, the patient received further treatment in the oncology department, and was reviewed in our hospital 3 months, 6 months, and 1 year after surgery. No significant postoperative complications were found, and the postoperative recovery was satisfactory.

## Discussion

OMFS is usually performed under GA. However, due to the increase of elderly patients and the complexity of patients’ conditions, anesthesiologists are faced with many problems in anesthesia management. For older patients, most anesthesiologists and surgeons believe that surgery is safer if it can be performed under regional or local infiltration anesthesia rather than GA (13). For older patients, GA requires a greater degree of preoperative preparation, increasing the complexity of care and higher costs and complications. On the contrary, RA has fewer complications and has the advantages of technical convenience, safety and cost effectiveness (14). RA increases the safety of surgery in elderly patients by reducing drug doses and side effects compared to GA, while still providing adequate analgesia.

To date, the effectiveness of CPB in various surgical procedures has been reported (7, 15–17). In the last few years, ultrasound-guided surface, intermediate, and deep CPB have emerged, improving efficacy and safety in several disciplines (12, 18, 19). SCPB has been used in a variety of head and neck surgeries and provides effective pain control and adequate regional anesthetic effects (20, 21). SCPB technology ensures the safety and patient comfort of performing surgery in the area around the neck and jaw (6). As Kanthan has seen, SCPB, in combination with other conventional nerve block anesthesia in the jaw region, provides excellent anesthesia and analgesia during skin incisions and tissue incision (6).

A thorough understanding of SCPB’s regional anatomy, markers, and application of correct techniques is essential for achieving good clinical anesthesia results. The cervical plexus (CP) is a network of nerve fibers formed by the combination of the anterior branches of the first to the fourth cervical nerve, and sometimes the anterior branches of the fifth cervical nerve. CP is located in the deep square of the sternocleidomastoid muscle, in front of the prevertebral muscle and the middle scalene muscle. The anterior branches of C2 to C4 form CP (C1 is the motor root component and is not anesthetized by SCPB). CP is divided into superficial cervical plexus (SCP), deep cervical plexus (DCP) and communicating branches. The SCP branches emerge from the posterior margin of the sternocleidomastoid muscle, forming four nerves to innervate the superficial structures of the neck, head, shoulders, and corresponding skin in the region. The four sensory nerve branches of the SCP are the minor occipital nerve, the major auricular nerve, the transverse cervical nerve, and the supraclavicular nerve. The minor occipital nerve, the major auricular nerve, and the transverse cervical nerve originate from C2 and C3. The minor occipital nerve climbs up to the posterior edge of the sternocleidomastoid muscle to innervate the area behind the ear. The great auricular nerve is distributed from the surface of the ear to the mandibular region and provides sensation to the surface structure of the parotid gland region. The nerve of the neck transversal across the sternocleidomastoid muscle facing forward, after penetrating the platysma muscle divides into anterior and posterior branches, distributed in the skin of the neck. It provides sensation from the sternal region to the mandibular region. Branches of the supraclavicular nerve come from C3 and C-4 and pass through the platysma muscle to the clavicle and shoulder, to the chest wall at the level of the lower second rib, to the acromioclavicular joint, and to the sternoclavicular joint. The transverse cervical and supraclavicular nerves provide sensation directly to the trapezius muscle. Local anesthetics can easily block all the nerves that make up the SCP. The branches produced by DCP innervate deep structures of the neck, diaphragm, and anterior neck muscles. In addition, many branches of the cervical plexus are connected to neighboring nerves, as well as to the X, XI, and XII cranial nerves (6). The ends of these nerves are located in the interfacial region below the sternocleidomastoid muscle (SCM) (19, 20). Thus, the superficial branches of the cervical plexus can be easily anesthetized by injecting anesthetic beneath the SCM.

With the development of ultrasound technology, new ultrasound-guided cervical plexus blocks have been introduced into various disciplines (e.g., emergency anesthesia and analgesia, thyroid surgery) (12, 22). In a recent study, Saranteas et al. successfully achieved deep CPB in patients undergoing thyroid surgery using ultrasound markers such as sternocleidomastoid muscle, prevertebral fascia, and ultrasound-recognized cervical transverse process (22). Herring et al. reported the successful use of ultrasound-guided SCPB in emergency anesthesia and analgesia to treat pain associated with clavicle fractures and lateral neck injuries (12). Targeting the superficial branch of CP at the midpoint of the posterior margin of the sternocleidomastoid muscle, SCPB can be routinely performed with ultrasound-guided subcutaneous injection techniques. So far, only two articles have reported the use of CPBA under ultrasound guidance in OMFS (11, 23). Perisanidis et al. studied the application of ultrasound-guided cervical middle and deep plexus block in OMFS (11). Yamaguchi et al. reported a case of right maxillary partial resection and neck lymph node dissection using bilateral ultrasound-guided maxillary (V2) nerve block combined with right SCPB (23). Although the analgesic effect of deep CPB is superior to superficial or intermediate CPB, it is more technically demanding and may be associated with more serious complications (such as intravascular, epidural, or subarachnoid injections), as well as respiratory complications associated with phrenic nerve paralysis (15). Using appropriate markers, one or more branches of the SCPB can be blocked, depending on the surgery performed in the neck and head area. Unlike the middle and deep cervical plexus block (DCPB), SCPB is easy to master (24). Ultrasound-guided block can improve anesthesia block efficiency, reduce anesthesia puncture injury, and further improve the accuracy and safety of SCPB (25).

In this paper, we report for the first time the application of ultrasound-guided SCPB anesthesia combined with LIA in OMFS. We observed that SPCB combined with LIA was highly effective in providing anesthesia in the neck area and controlling pain during and during the first 24 hours after anesthesia in all patients, with no block-related complications. In addition, the cost of patient care can be reduced due to the shorter recovery time and shorter surgical time. Intraoperative verbal communication between the surgeon and patient, including reassurance or advance notification of the surgical procedure (e.g., cauterization), can effectively relieve the patient’s tension, anxiety, panic and fear. In addition, patients with persistent cough were given antitussive agents before surgery for the smooth operation. Intraoperatively, all patients were breathing spontaneously, took deep breaths on command and cooperated well.

We believe that ultrasound-guided SCPB combined with LIA is a safe and effective surgical anesthesia method in some oral and maxillofacial surgeries when the patients’ general condition is poor, especially the elderly patients who have a risk for GA, in order to give them an operation opportunity, restore their health and save their lives.

## Conclusion

Ultrasound-guided SCPB combined with LIA is a simple, safe and effective anesthesia method when some patients with specific diseases, especially elderly patients with poor general condition who are at risk of general anesthesia. Ultrasound-guided SCPB combined with other LIA provides adequate RA for maxillofacial region, which has a high success rate, low complications, high patient acceptance and negligible morbidity and mortality. It can be used as an alternative to GA in certain types of maxillofacial surgery. There were no poorly documented complications in our case, and more randomized trials are needed to compare the effectiveness and complication rate of SPCB combined with LIA in OMFS.

In summary, when elderly patients are at high medical risk for GA, ultrasound-guided SPCB combined with LIA may be a suitable alternative.

## Data Availability

The original contributions presented in the study are included in the article/supplementary material. Further inquiries can be directed to the corresponding author.

## References

[1] Liao P Yan B Wang C and Lei P. Telomeres: dysfunction, maintenance, aging and cancer. Aging Dis. (2023). doi: 10.14336/AD.2023.1128 PMC11567242 38270117

[2] Feng Z Glinskaya E Chen H Gong S Qiu Y Xu J. Long-term care system for older adults in China: policy landscape, challenges, and future prospects. Lancet. (2020) 396:1362–72. doi: 10.1016/S0140-6736(20)32136-X 34338215

[3] Lobanov-Rostovsky S He Q Chen Y Liu Y Wu Y Liu Y. Growing old in China in socioeconomic and epidemiological context: systematic review of social care policy for older people. BMC Public Health. (2023) 23:1272. doi: 10.1186/s12889-023-15583-1 37391766 PMC10311713

[4] Bhushan S Huang X Duan Y and Xiao Z. The impact of regional versus general anesthesia on postoperative neurocognitive outcomes in elderly patients undergoing hip fracture surgery: A systematic review and meta-analysis. Int J Surg. (2022) 105:106854. doi: 10.1016/j.ijsu.2022.106854 36031067

[5] Masters RD Castresana EJ Castresana MR. Superficial and deep cervical plexus block: technical considerations. Aana J. (1995) 63:235–43. 7631578

[6] Kanthan RK. The use of superficial cervical plexus block in oral and maxillofacial surgical practice as an alternative to general anesthesia in selective cases. Ann Maxillofac Surg. (2016) 6:4–8. doi: 10.4103/2231-0746.186120 27563598 PMC4979341

[7] Suresh S Templeton L. Superficial cervical plexus block for vocal cord surgery in an awake pediatric patient. Anesth Analg. (2004) 98:1656–7. doi: 10.1213/01.Ane.0000114073.26682.A1 15155321

[8] Pandit JJ McLaren ID Crider B. Efficacy and safety of the superficial cervical plexus block for carotid endarterectomy. Br J Anaesth. (1999) 83:970–2. 10700811

[9] Saxe AW Brown E Hamburger SW. Thyroid and parathyroid surgery performed with patient under regional anesthesia. Surgery. (1988) 103:415–20. 3353856

[10] Shteif M Lesmes D Hartman G Ruffino S Laster Z. The use of the superficial cervical plexus block in the drainage of submandibular and submental abscesses–an alternative for general anesthesia. J Oral Maxillofac Surg. (2008) 66:2642–5. doi: 10.1016/j.joms.2008.05.365 19022150

[11] Perisanidis C Saranteas T Kostopanagiotou G. Ultrasound-guided combined intermediate and deep cervical plexus nerve block for regional anaesthesia in oral and maxillofacial surgery. Dentomaxillofac Radiol. (2013) 42:29945724. doi: 10.1259/dmfr/29945724 22933534 PMC3699012

[12] Herring AA Stone MB Frenkel O Chipman A Nagdev AD. The ultrasound-guided superficial cervical plexus block for anesthesia and analgesia in emergency care settings. Am J Emerg Med. (2012) 30:1263–7. doi: 10.1016/j.ajem.2011.06.023 22030184

[13] Bodenham AR Howell SJ. General anaesthesia vs local anaesthesia: an ongoing story. Br J Anaesthesia. (2009) 103:785–9. doi: 10.1093/bja/aep310 19918020

[14] Teulières M Berard E Marot V Reina N Ferre F Minville V. A quadruple peripheral nerve block outside the OR for anterior cruciate ligament reconstruction reduces the OR occupancy time. Knee Surg Sports Traumatol Arthrosc. (2023) 31:2917–26. doi: 10.1007/s00167-022-07246-2 36469051

[15] Pandit JJ Satya-Krishna R Gration P. Superficial or deep cervical plexus block for carotid endarterectomy: a systematic review of complications †. Br J Anaesthesia. (2007) 99:159–69. doi: 10.1093/bja/aem160 17576970

[16] Aunac S Carlier M Singelyn F De Kock M. The analgesic efficacy of bilateral combined superficial and deep cervical plexus block administered before thyroid surgery under general anesthesia. Anesth Analg. (2002) 95:746–50. doi: 10.1097/00000539-200209000-00039 12198064

[17] Pintaric TS Hocevar M Jereb S Casati A Novak Jankovic V. A prospective, randomized comparison between combined (deep and superficial) and superficial cervical plexus block with levobupivacaine for minimally invasive parathyroidectomy. Anesth Analg. (2007) 105:1160–3. doi: 10.1213/01.ane.0000280443.03867.12 17898405

[18] Saranteas T Paraskeuopoulos T Anagnostopoulou S Kanellopoulos I Mastoris M Kostopanagiotou G. Ultrasound anatomy of the cervical paravertebral space: a preliminary study. Surg Radiol Anat. (2010) 32:617–22. doi: 10.1007/s00276-010-0621-9 20082079

[19] Kefalianakis F Koeppel T Geldner G Gahlen J. Carotid-surgery in ultrasound-guided anesthesia of the regio colli lateralis. Anasthesiologie Intensivmedizin Notfallmedizin Schmerztherapie: AINS. (2005) 40:576–81. doi: 10.1055/s-2005-870377 16252218

[20] Kim J-S Ko JS Bang S Kim H Lee SY. Cervical plexus block. Korean J Anesthesiol. (2018) 71:274–88. doi: 10.4097/kja.d.18.00143 PMC6078883 29969890

[21] Pandit JJ Dutta D Morris JF. Spread of injectate with superficial cervical plexus block in humans: an anatomical study † †Presented in part at the Anaesthetic Research Society Meeting, Leeds, July 6, 2001. Br J Anaesthesia. (2003) 91:733–5. doi: 10.1093/bja/aeg250 14570798

[22] Saranteas T Kostopanagiotou G Anagnostopoulou S Mourouzis K Sidiropoulou T. A simple method for blocking the deep cervical nerve plexus using an ultrasound-guided technique. Anaesthesia Intensive Care. (2011) 39:971–2. 21970150

[23] Yamaguchi A Kojima Y Hirabayashi K. Ultrasound-guided maxillary nerve block and superficial cervical plexus block during surgery for maxillary Malignancy: A case report. Anesth Prog. (2023) 70:88–90. doi: 10.2344/anpr-70-02-07 37379090 PMC10328195

[24] Kesisoglou I Papavramidis TS Michalopoulos N Ioannidis K Trikoupi A Sapalidis K. Superficial selective cervical plexus block following total thyroidectomy: a randomized trial. Head Neck. (2010) 32:984–8. doi: 10.1002/hed.21286 19953610

[25] Goulart TF Araujo-Filho VJF Cernea CR Matos LL. Superficial cervical plexus blockade improves pain control after thyroidectomy: A randomized controlled trial. Clinics (Sao Paulo). (2019) 74:e605. doi: 10.6061/clinics/2019/e605 31531572 PMC6735272

